# SeArcH schemes for Approximate stRing mAtching

**DOI:** 10.1093/nargab/lqaf025

**Published:** 2025-03-18

**Authors:** Simon Gene Gottlieb, Knut Reinert

**Affiliations:** Informatik & Mathematik, Freie Universität Berlin, Takustraße 9, 14195 Berlin, Germany; Informatik & Mathematik, Freie Universität Berlin, Takustraße 9, 14195 Berlin, Germany; Efficient Algorithms for Omics Data Group, Max-Planck-Institute for Molecular Genetics, Berlin 14195, Germany

## Abstract

Finding approximate occurrences of a query in a text using a full-text index is a central problem in stringology with many applications, especially in bioinformatics. The recent work has shown significant speed-ups by combining bidirectional indices and employing variations of *search schemes*. Search schemes partition a query and describe how to search the resulting parts with a given error bound. The performance of search schemes can be approximated by the *node count*, which represents an upper bound of the number of search steps. Finding *optimum search schemes* is a difficult combinatorial optimization problem that becomes hard to solve for four and more errors. This paper improves on a few topics important to search scheme based searches: First, we show how search schemes can be used to model previously published approximate search strategies such as suffix filters, 01*0-seeds, or the pigeonhole principle. This unifies these strategies in the search scheme framework, makes them easily comparable and results in novel search schemes that allow for any number of errors. Second, we present a search scheme construction heuristic, which is on par with optimum search schemes and has a better node count than any known search scheme for equal or above four errors. Finally, using the different search schemes, we show that the node count measure is not an ideal performance metric and therefore propose an improved performance metric called the *weighted node count*, which approximates a search algorithm’s run time much more accurately.

## Introduction

Finding approximate occurrences of a string in a large text is a fundamental problem in computer science with numerous applications, especially in bioinformatics. In this paper, we will address the problem for Hamming distance as well as for edit distance.

More formally, the approximate string matching (ASM) problem considered in this paper is defined as follows: Given an error number *k*, a string, here referred to as a *queryQ*, and a text *T*, composed of characters from an alphabet of size |Σ|, find substrings of the text whose distance to the query is at most *k*.

Solving the ASM problem has become especially important in bioinformatics due to the advances in sequencing technology during the last years. The mainstream second-generation sequencing techniques like Illumina produce reads of length 150–250 with an error rate of about 1%, mostly substitutions caused by the sequencing technology.

Other sequencing technologies, e.g. Pacific Bioscience or Oxford Nanopore, produce much longer reads but with a higher error rate (in the range of up to 15%) containing substitutions, insertions, and deletions. A standard problem is to map the reads back to a reference genome while taking into account the errors introduced by the sequencing technology as well as those caused by biological variations, such as SNPs or small structural variations. Such a problem is almost always modeled as the ASM problem for Hamming or edit distance.

There are two main algorithmic strategies to address the ASM problem for large inputs, including a long reference text and/or a high number of reads: *filtering* and *indexing*.

In this work, we focus on the indexing approach. Here, the main idea is to preprocess the reference text, the set of reads, or both, more intricately.

Such preprocessing into *full-text string indices* has the benefit that we do not have to scan the whole reference but can conduct queries much faster at the expense of larger memory consumption. String indices currently used are the suffix array [1], enhanced suffix array [2], and affix arrays [3, 4], as well as the FM-index [5], a data structure based on the Burrows–Wheeler Transform (BWT) [6] and some auxiliary tables. For an in-depth discussion see [7]. Such indices are used to compute exact matches between a query and a text as a subroutine in backtracking approaches. For the ASM problem for Hamming or edit distance, the existing algorithms all have exponential complexity in *k* (e.g. [8, 9]), and are hence only suited for small *k*.

Lam *et al.* [10] introduced an idea based on *bidirectional* FM indices to speed up ASM for Hamming distance. For the cases *k* = 1 and 2, they partitioned the read into *k* + 1 equal parts and argued that performing approximate matching on a certain combination of these parts in a bidirectional index amounts to *faster* approximate matching of the whole read. This combination is such that all possible *error configurations*, i.e., all possible distributions of *k* mismatches among the parts, are covered. The main idea behind improved speed is that a bidirectional index does not have to start the search from the beginning or end of the read but from any of the parts. Therefore, we can start the search from a middle part and then expand it to the left and right into adjacent parts in any order we like. By choosing multiple appropriate orderings of parts for this purpose, we can perform a much faster ASM compared to a unidirectional search. By enforcing exact or near-exact searches on the first parts of the partition, we significantly reduce the number of backtrackings or spurious results. By using different orderings of parts, we ensure that all possible error configurations are covered.

Kucherov *et al.* [11] formalized and generalized this idea by defining the concept of *search schemes*. Assume a read can be partitioned into a given number of parts, denoted by *p*. The parts are indexed from left to right. A search scheme $\mathcal {S}=\lbrace (\pi _s,L_s,U_s)| s=1, \ldots , S\rbrace$ is a collection of *S* searches, where each search *s* is designated by a triplet (π_*s*_, *L*_*s*_, *U*_*s*_). π_*s*_ is a permutation of 1, …, *p* and denotes the order in which the parts of the partition are searched in search *s*. *L*_*s*_ and *U*_*s*_ are denoting the accumulated lower and upper bounds of allowed errors for each part.

Kianfar and colleagues [12] in cooperation with our group, proposed a method to compute *optimal search schemes* for Hamming distance under certain constraints by minimizing the overall number of edges in the backtracking trees of the search schemes. Their method is based on solving a Mixed-Integer Linear Program (ILP), which is costly. Hence, they can only compute solutions for up to four errors in reasonable time and only guarantee for 1 and 2 errors to be optimal. This paper mentions that Vrolands *et al.* [9] 01*0 seeds can be reformulated as search schemes.

Renders *et al*. [13] implemented improvements of the applied search schemes into their tool Columba. Instead of backtracking single error configurations, they use a banded matrix to track multiple error configuration. By leaving out well-defined paths through the matrix the number of search steps is significantly decreased. To our knowledge and experience this is currently the fastest implementation to execute search schemes. Additionally, they benchmark search schemes based on the pigeonhole principle and 01*0 seeds for up to four errors.

In their latest work [14], Renders *et al.* explore the possibility of automated design of efficient search schemes. They implement a new ILP formulation that is capable of generating search schemes for up to *k* = 7 errors. They show that search schemes generated with their tool *hato* and executed with *Columba* outperform existing tools for lossless approximate search.

Searching in a bidirectional index using any given, valid search scheme is already implemented in our library *SeqAn* [15] for Hamming and edit distance. Hence, it is attractive to investigate methods to construct search schemes for larger *k*. This fact and the shortcomings of the method of Kianfar *et al.* inspired the work we present in this paper.

We show how to cleverly formulate suffix filters [8], 01*0 seeds [9], and pigeonhole filtering in terms of search schemes and compare the performances of all methods using the Columba tool [13]. We propose a novel metric *weighted node code* to evaluate search schemes that correlates much better to their actual performance than the *node count* measure used in [12].

Finally, we will underpin these claims by collecting benchmark for all search schemes with Columba [13] and compare the run times to the node count and our newly introduced weighted node count. Supporting that the weighted node count is a good performance indicator of search schemes.

## Material and methods

### Nomenclature

In this section, we introduce fundamental notations and definitions. We use 0-based indices, which should be interpreted as *offsets* from the first entry. A string is denoted as *A* = *a*_0_*a*_1_…*a*_|*A*| − 1_ where |*A*| denotes the length and *a*_*i*_ refers to a single character of the string. An infix can be denoted as *A*_*i*, *j*_ = *a*_*i*_*a*_*i* + 1_…*a*_*j* − 1_. The approximate search of a query *Q* = *q*_0_, *q*_1_, …*q*_|*Q*| − 1_ inside a text *T* = *t*_0_, *t*_1_, …, *t*_*n* − 1_ is defined as finding a set of substrings in $\lbrace t_{s_0, e_0}, t_{s_1, e_1}, \ldots \rbrace$ that are similar under some metric *dist*( ·, ·), such that $dist( t_{s_i, e_i}, Q) \le k$ where *k* is the number of errors for the distance measure. For metrics, we exclusively use either Hamming distance *dist*_*ham*_ or edit distance *dist*_*edit*_.

A bidirectional index as the 2FM-Index allows us to search for a query *Q* by starting at any point in our query and extending it to the left or right until our full query is covered. In a broad sense, a search scheme describes how to extend a query step by step and how many errors are expected.

Assume the query *Q* is divided into *p* parts *P* = (*P*_0_, …, *P*_*p* − 1_) such that the concatenation of the parts equals *Q*. A *search scheme* is defined as a set of *searchesS* = {*s*_0_, *s*_1_, …, *s*_|*S*| − 1_}. Each search is defined as a triplet *s*_*i*_ = (π_*i*_, *L*_*i*_, *U*_*i*_). The *search order* of parts is defined by a permutation π = (π_0_, …, π_*p* − 1_) of {0, …, *p* − 1}. To work with bidirectional indices, e.g. a 2FM-Index, it is also required that π fulfills the *connectivity property*, which states that each π_*i*_ has to be one larger than the maximum of the previous π_*k*_ or one smaller than the minimum. Further on, let *L*_*i*_ = (*L*_*i*, 0_, …, *L*_*i*, *p* − 1_) denote the minimal accumulated error and *U*_*i*_ = (*U*_*i*, 0_, …, *U*_*i*, *p* − 1_) denote the maximal accumulated error while searching the part in the order of π. Then it must hold that the values in *U*_*i*_ and *L*_*i*_ are monotonously increasing and the upper bound is larger or equal to the lower bound *U*_*i*, *j*_ ≥ *L*_*i*, *j*_, ∀*j*. Search schemes that fulfill those two requirements for all searches can be used for searching in a bidirectional index and are hence called *valid*. In the following, we will drop the search index *i* from *L*_*i*_ and *U*_*i*_ and write *L* and *U* if the context is clear. The expression *L* = (*x*, *y*, *z*, …) will be shortened to *L* = *xyz* to increase readability.

An *error configuration* is a distribution of the *k* errors over the parts and is denoted by (*e*_0_, …, *e*_*p* − 1_) with $\sum _{i=0}^{p-1} e_i=k$. If a search scheme covers all possible error configurations, it is called *complete*. Additionally, if each error configuration is covered by only one search, it is called *non-redundant*. In the case of hamming distance a *complete* and *non-redundant* search scheme will lead to each match being found exactly once. Contrary, edit distance based searches can find the same position with different alignments.

An example of a search scheme can be seen in Fig. 2, where the standard pigeonhole principle for two errors is expressed as a valid and complete search scheme with three searches. It can be seen that all error configurations are covered, albeit some by several searches. Hence, the search scheme is redundant. This opens the door for devising an improved version of the pigeonhole search scheme that we will introduce in the Subsection *Formulating search strategies as search schemes*.

**Figure 1.**
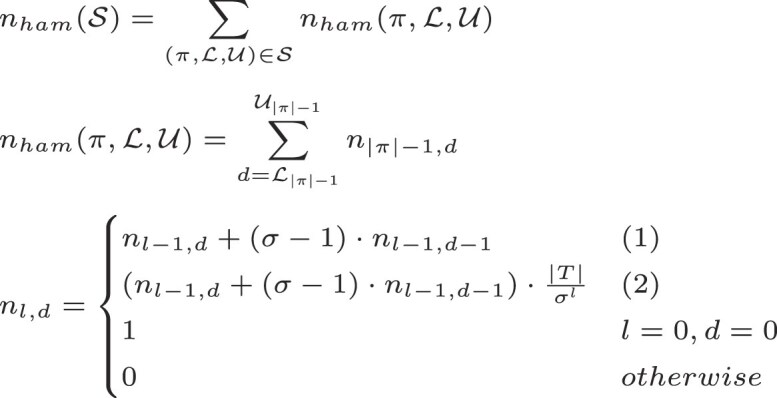
Conditions: (1) $\mathcal {L}_l \le d \le \mathcal {U}_l \wedge 1 \le l \le \lceil \log _{\sigma } |T| \rceil$ (2) $\mathcal {L}_l \le d \le \mathcal {U}_l \wedge l >\lceil \log _{\sigma } |T| \rceil$ The depiction of the weighted node count for edit distance can be found in the appendix.

**Figure 2.**
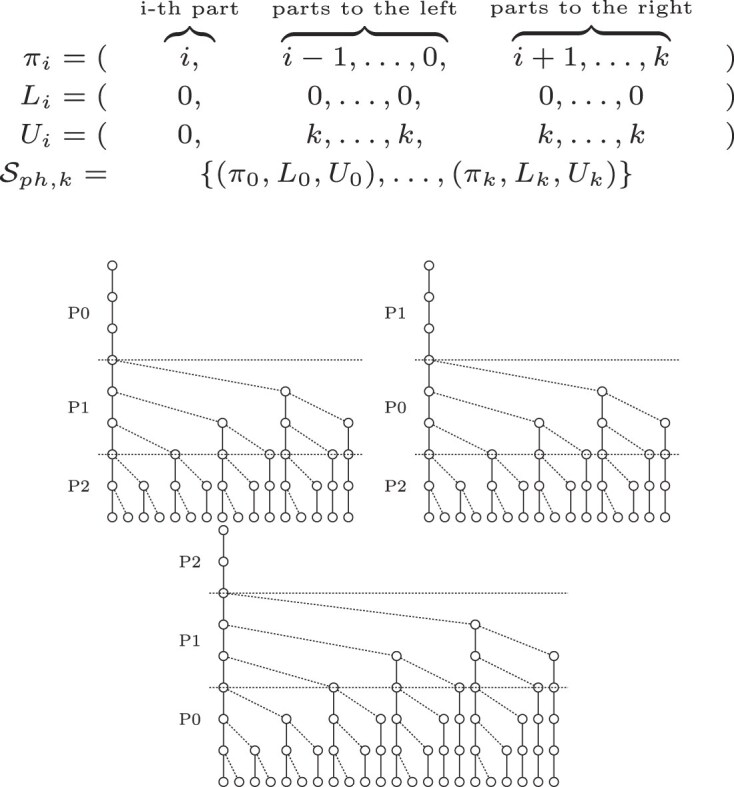
Pigeonhole principle expressed as a search scheme *S* = {*s*_0_ = (012, 000, 022), *s*_1_ = (102, 000, 022), *s*_2_ = (210, 000, 022)} with *k* = 2, query length |*Q*| = 8 and an alphabet of |Σ| = 2 for *dist*_*ham*_. Following an edge down symbolizes a match between query and text. Branching to the right indicates a mismatch. The first search covers the error configurations {(0, 0, 0), (0, 0, 1), (0, 1, 0), (0, 1, 1), (0, 0, 2), (0, 2, 0)}, the second search covers {(0, 0, 0), (1, 0, 0), (1, 0, 1), (0, 0, 1), (0, 0, 2), (2, 0, 0)} and the third search covers {(0, 0, 0), (1, 0, 0), (0, 1, 0), (1, 1, 0), (0, 0, 2), and (0, 2, 0)}.

### Node count

The quality of a search scheme can be measured by counting the number of extensions applied to a pattern, which equals the number of search steps in the 2FM-Index. This measure is also referred to as the *node count* and was introduced by Kianfar *et al.* [12]. To compute the node count of a search scheme we have to assume a distance metric, pattern length, and the alphabet size. From the pattern length, we can compute vectors $\mathcal {L}$ and $\mathcal {U}$ which are similar to *L* and *U* but are inflated to the length of our pattern. For search *s*_0_ in Fig. 2, the bounds *L* = 000, *U* = 022 are inflated to $\mathcal {L}^{(edit)}=000\, 000\, 00$, $\mathcal {U}^{(edit)}=000\, 222\, 22$, or $\mathcal {L}^{(ham)}=000\, 000\, 00$, $\mathcal {U}^{(ham)}=000\, 122\, 22$.

### Weighted node count

However, the node count has a few shortcomings as a performance indicator for search schemes. This becomes especially noticeable when comparing run times of backtracking to other formulated search schemes when using higher error rates.

We introduce an improved *weighted node count*, which takes into consideration that not all children of every node can be visited. Depending on the size of the indexed text *T*, it is guaranteed that at a certain depth the search will not visit every node. At depth *l*, the number of possible backtracking branches is $\text{#branches} = |\Sigma |^l$. A concrete reference *T* can have at most |*T*| − *l* different *l-mers*. Hence, below level *l* = ⌈log_|Σ|_|*T*|⌉ not all branches of the search scheme will be visited. Using this reasoning, we change the computation of the node count. From level *l* on, the maximal number of nodes being visited is lower than the total number of nodes. With this, the weighted node count for Hamming distance can be computed as seen in Fig. 1.

The weighted node count for edit distance looks similar but has additional branches. We included the equation in the appendix.

### Formulating search strategies as search schemes

To formulate published search strategies into search schemes, we need to determine a method to generate $\mathcal {S}=\lbrace (\pi _s, L_s, U_s)| s=1, \ldots , S\rbrace$ for an allowed number of errors. We have to ensure that the search scheme is valid and complete. All our illustrations are for the Hamming distance, but the schemes are equally valid for the edit distance. In the following, we will describe several search schemes based on different strategies:

-
$\mathcal {S}_{ph, k}$ : Pigeonhole based with *k* errors.
-
$\mathcal {S}_{ph-opt, k}$ : Optimized pigeonhole based with *k* errors.
-
$\mathcal {S}_{sf, k}$ : Suffix filter based with *k* errors.
-
$\mathcal {S}_{01*0,k }$ : 01*0 seeds based with *k* errors.
-
$\mathcal {S}_{01*0-opt, k}$ : Optimized 01*0 seeds based with *k* errors.
-
$\mathcal {S}_{H, k, p}$ : Our custom heuristic to construct search schemes with *k* errors and *p* parts.

Later on, we will compare these to additional search schemes:

-
$\mathcal {S}_{bt, k}$ : Backtracking scheme with a single search.
-
$\mathcal {S}_{OSS,k }$ : Optimum Search Schemes provided by Kianfar *et al.* [12].
-
$\mathcal {S}_{kianfar,k }$ : Optimum search scheme that are split into *k* + 1 parts, also described by Kianfar *et al.* [12].
-
$\mathcal {S}_{kuch, k, k+1}$ : Splitting the search into *k* + 1 parts. For *k* ∈ {1, 2, 3, 4} described by Kucherov *et al.* [11].
-
$\mathcal {S}_{kuch, k, k+2}$ : Splitting the search into *k* + 2 parts. For *k* ∈ {1, 2, 3, 4} described by Kucherov *et al.* [11].
-
$\mathcal {S}_{hato, k}$ : Search schemes provided by Renders *et al.* [14].

All used search schemes with concrete numbers are listed in the supplementary file in the *Search Schemes* section.

#### Pigeonhole strategy

From the pigeonhole principle, we can derive that a query with *k* errors divided into *k* + 1 parts will imply that at least one of the parts has to have zero errors. By performing *k* + 1 searches where each search starts with a different part with zero errors, we can create a valid and complete search scheme. An example of this search scheme is shown in Fig. 2.

The above search scheme for the pigeonhole principle can be improved with a tighter lower and upper bound. For example, the first search of $\mathcal {S}_{ph, k}$ finds all substrings with zero errors in the first part. The second and further searches can avoid searching for those results. Careful inspection shows, that for search *i*, we could require at least one error on each part to the left of part *i*. These parts will have been found with zero errors by previous searches. To achieve this, we need to increase the bounds when searching to the left of part *i* (i.e. parts *i* − 1 to 0). For these parts, the lower bound can be set to 1 for part *i* − 1 and increased until value *i* is reached for part 0.

Regarding the upper bounds, we can avoid the search for certain values for the parts to the left of *i*. This is achieved by decreasing the upper bound to *k* − *i* + 1 for the (*i* − 1)th part and allowing one additional error for every next part to the left until *k* is reached.

**Figure 3.**
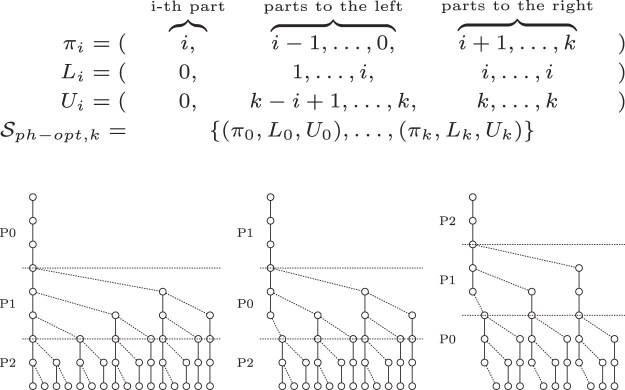
An optimized version of the pigeonhole principle expressed as a search scheme *S* = {(012, 000, 022), (102, 011, 022), and (210, 012, 012)} with *k* = 2, query length |*Q*| = 8 and an alphabet of *σ* = 2. The first search covers the error configurations {(0, 0, 0), (0, 0, 1), (0, 1, 0), (0, 1, 1), (0, 0, 2), and (0, 2, 0)}, the second search covers {(1, 0, 0), (1, 0, 1), and (2, 0, 0)} and finally the third search covers {(1, 1, 0)}.

The resulting search scheme depicted in Fig. 3 has a lower node count and the error configurations covered by each of the searches are disjoint, in contrast to the standard pigeonhole principle depicted in Fig. 2. Optimized pigeonhole-based search schemes with higher errors *k* > 2 have redundant searches.

#### Suffix filter

The suffix filter can be seen as an extension of the pigeonhole principle. Assume we have a factorization of the query into *k* + 1 factors. Then according to Kärkkäinen and Na [8], there must exist a *strong match* of a factor suffix, which means the sum of the errors of *s* factors is smaller than *s*. We can model this as a search scheme, by first searching a part *i* with zero errors, then extending the search to the right as long as the strong match criterion is met, and finally extending the parts to the left.

**Figure 4.**
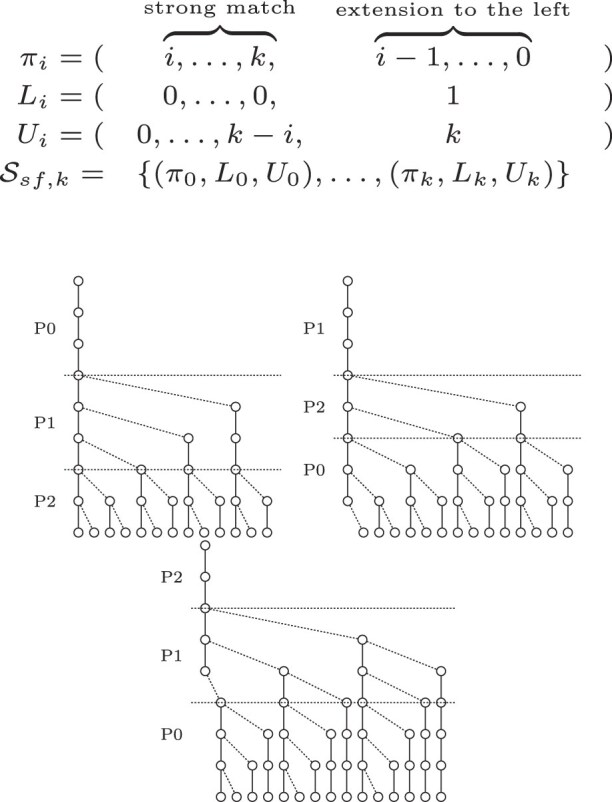
Suffix filter expressed as a search scheme with *k* = 2, query length |*Q*| = 8, and an alphabet of *σ* = 2. The first search covers the error configurations {(0, 0, 0), (0, 0, 1), (0, 1, 0), (0, 1, 1), and (0, 0, 2)}, the second search covers {(1, 0, 0), (1, 0, 1), (2, 0, 0), and (0, 1, 0)} and finally the third search covers {(1, 1, 0), (0, 2, 0), and (0, 1, 0)}.

For our small example, the suffix filter search scheme is depicted in Fig. 4. Note the difference in the error configurations when comparing to the optimized pigeonhole scheme depicted in Fig. 3. For these parameters, the suffix filter search scheme is redundant.

#### 01*0 seeds

The 01*0 seeds strategy of Vroland *et al.* [9] follows a formulation that is stronger than the pigeonhole principle. It states that if a read has *k* errors and is divided into *k* + 2 parts then there exists at least 1 group of adjacent parts where the first and last part have zero errors and all parts in between have exactly one error. In the original formulation, 01*0 seeds are found using a unidirectional FM-Index and extended and verified in text. In contrast to this strategy, we formulate the complete search of the query as a search scheme. Let *j* be the number of parts that match with exactly one error. Then we can formulate the strategy with an ordering that matches part *i* with 0 errors, then the next *j* parts with exactly 1 error, then part *i* + *j* + 1 again with 0 errors. The remaining errors are distributed over the remaining parts. Formally the search scheme reads as:


\begin{eqnarray*} \begin{array}{llllllll}\mathcal {S}_{01*0, k} =\lbrace & (\pi _{0,0},& L_{0,0},& U_{0,0}),& \ldots ,& (\pi _{0,k}, & L_{0,k}, & U_{0,k}),\\ & (\pi _{1,0},& L_{1,0},& U_{1,0}),& \ldots ,& (\pi _{1,k-1},& L_{1,k-1},& U_{1,k-1})\\ & \ldots & & & & & &\\ & (\pi _{k,0},& L_{k,0},& U_{k,0})\rbrace & & & & \end{array} \end{eqnarray*}

**Figure 5.**
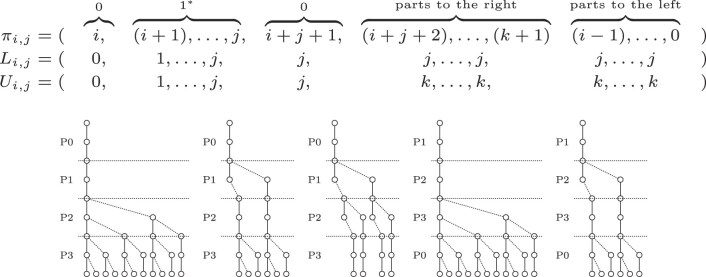
01*0 seeds expressed as a search scheme with *k* = 2, query length |*Q*| = 8, and an alphabet of *σ* = 2.

**Figure 6.**
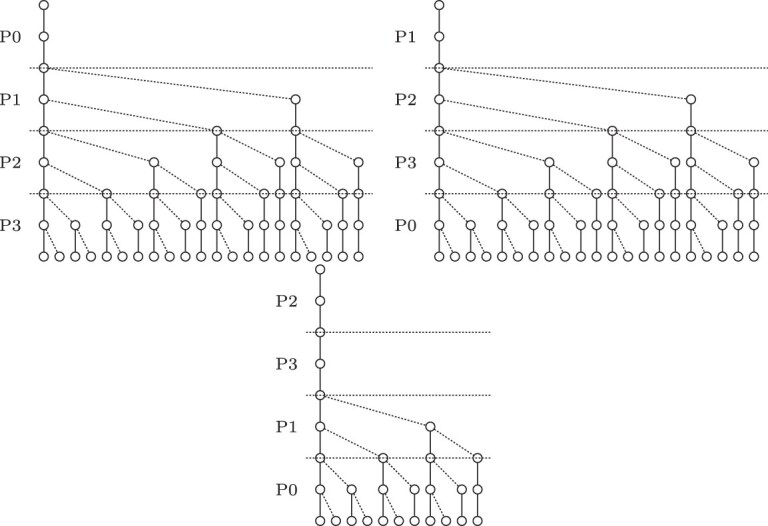
Optimized 01*0 seeds expressed as a search scheme with *k* = 2, query length |*Q*| = 8, and an alphabet of *σ* = 2.

The search scheme $\mathcal {S}_{01*0, k}$ produces searches with the same order of parts. This enables merging searches, optimizing the number of searches. We consider this an optimized 01*0 seeds strategy which contains exactly *k* searches. A disadvantage of merging searches is that it creates additional search paths, that did not exist in the original $\mathcal {S}_{01*0, k}$ search scheme. In Fig. 5, a $\mathcal {S}_{01*0, k}$ search scheme is depicted. It consists of five searches of which multiple searches search the parts in exactly the same order with different error bounds. The optimized scheme $\mathcal {S}_{01*0_opt, k}$ with fewer searches but more nodes is depicted in Fig. 6.


\begin{eqnarray*} \pi _{opt,i} &=& \pi _{i, 0}\\ L_{opt,i} &=& \min (L_{i, j})\\ U_{opt,i} &=& \max (U_{i, j})\\ \mathcal {S}_{01*0-opt, k} &=& \lbrace (\pi _{opt, i}, L_{opt, i}, U_{opt, i}) | 0 \le i \le k\rbrace \end{eqnarray*}

We evaluate the proposed search schemes in Subsection *Evaluation*. We compare the theoretical performance by computing the node count and measure the actual run time.

### Our search scheme heuristic

In this subsection, we provide a description on how to construct a search scheme $\mathcal {S}_{H, k, p} = \lbrace (\pi _0, L_0, U_0), \ldots )\rbrace$. Our goal is to construct search schemes with a low node count, which can be used for errors *k* ≥ 4 for which optimum search schemes are unknown. This algorithm does not guarantee to create optimum search scheme, we also do not know if the search schemes produced are *complete* or *valid*. Because of these limitations, we consider our method a heuristic for search scheme construction. However, we checked that the resulting search schemes are *valid* and *complete* for up to 15 errors. Additionally, our heuristic has the same node count as the known optimum search schemes and the lowest node count compared to all known search schemes for higher errors.

For our construction algorithm, we analyzed the known optimum search schemes, where we noticed that they seemingly have an internal structure. We will describe a parameterized construction which will reproduce these search schemes. The construction algorithm has two user defined parameters *k* and *p*. The parameter *k* indicates the allowed number of errors our scheme should cover. The parameter *p* controls the number of parts and must be chosen such that *p* > *k* (This is a limitation of our construction, it likely could be removed). To find a search schemes with a low node count one must iterate over different values of *p*. In all our test cases, *p* = *k* + 2 results in the lowest node count, with the exception of *k* ≤ 2 where *p* = *k* + 1 has the lowest node count.

From these two parameters, we need to derive the number of searches and for each search the order of parts as well as the lower and upper bound of each part. Computing the number of searches, order of parts and the lower bound is much easier than the upper bound. Starting with the number of searches, we fix these to *k* + 1.

The order of the parts for a search *i* is defined as follows:


\begin{eqnarray*} \pi _i = (\overbrace{i, \ldots , p-1}^{\text{extending right}},\overbrace{i-1, \ldots , 0}^{\text{extending left}}) \end{eqnarray*}

The lower bound of the parts are defined as follows:


\begin{eqnarray*} L_i = (\overbrace{0, \ldots , 0}^{\times (p-i-1)},\overbrace{k-i,\ldots ,k-i}^{\times (i+1)}) \end{eqnarray*}

The upper bound *U*_*i*_ requires an additional matrix *M*. The matrix *M* defines the maximum number of errors that are allowed in a part. The rows correspond to the searches, and each column corresponds to a part of the query:


\begin{eqnarray*} M &=& \begin{pmatrix}{\begin{array}{*{10}c}M_k\\ \hline M_b \end{array}} & M_t \end{pmatrix}\\ &=& \begin{pmatrix}\begin{array}{cccc}m_{0, 0} & \ & m_{0, k-1}\\ m_{1, 0} & \ldots & m_{1, k-1}\\ \ldots & & \ldots \\ m_{k-1, 0} & \ldots & m_{k-1, k-1}\\ \hline m_{k, 0} & \ldots & m_{k, k-1}\\ \end{array} & \begin{array}{ccc}m_{0, k} & \ldots & m_{0, p-1}\\ m_{1, k} & \ldots & m_{1, p-1}\\ \ldots & & \ldots \\ m_{k, k} & \ldots & m_{k, p-1} \end{array} \end{pmatrix} \end{eqnarray*}

The matrix *M* is split into three sub matrices called *M*_*k*_, *M*_*b*_, and *M*_*t*_. Each of these matrices have to satisfy additional constraints:


\begin{eqnarray*} \forall \; 0 \le i < k:\; m_{j, i} &=& (j+i \mod {k})\\ \forall \; 0 \le i < k:\; m_{k, i} &=& k\\ \forall \; i \ge k \wedge 0 \le j \le k:\; m_{j, i} &=& k-j\\ \forall j< i:\; & m_{i, j+1} \le m_{i, j}\\ \forall j > i:\; & m_{i, j-1} \le m_{i, j} \end{eqnarray*}

The first three conditions can be used to to generate submatrices *M*_*k*_, *M*_*b*_, and *M*_*t*_ respectively. The last two conditions are not automatically fulfilled after generating *M*. In an adjustment step, the first column *i* with an element *m*_*j*, *i*_ violating these condition has to be adjusted by permuting its elements. The elements *m*_0, *i*_ until *m*_*k* − 1, *i*_ without the element *m*_*i*, *i*_ will be permuted until a valid combination is found. (We do not know if this step will always terminate, in our tests it worked for *k* ≤ 1000.) This adjustment step has to be repeated until all columns fulfill these conditions. In a last step, the matrix M will be reordered to *M*_*ord*_ according to π:


\begin{eqnarray*} M_{ord} = \begin{pmatrix}m_{0, \pi _0[0]}& m_{0, \pi _0[1]}& \ldots & m_{0, \pi _0[p-1]}\\ m_{1, \pi _1[0]}& m_{1, \pi _1[1]}& \ldots & m_{1, \pi _1[p-1]}\\ \ldots & & \ldots & \ldots \\ m_{k, \pi _k[0]}& m_{k, \pi _k[1]}& \ldots & m_{k, \pi _k[p-1]} \end{pmatrix} \end{eqnarray*}

The upper bound of the parts can now be defined as follows, limiting the values to a maximum of *k*:


\begin{eqnarray*} U_i &=& ( \min (0 + M_{ord}[i][0], k),\\ && \quad \min (L_{i}[0] + M_{ord}[i][1], k),\\ &&\quad \ldots , \\ && \quad \min (L_i[p-2] + M_{ord}[i][p-1], k)) \end{eqnarray*}

**Figure 7.**
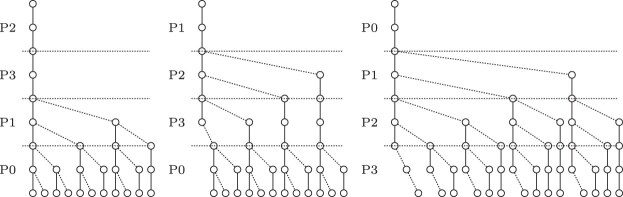
Search scheme generated by our construction algorithm. *S* = {*s*_0_ = (2310, 0000, 0022), *s*_1_ = (1230, 0011, 0112), and *s*_2_ = (0123, 0002, 0122)} with *k* = 2, query length |*Q*| = 8, 4-part-split and an alphabet of |Σ| = 2 for *dist*_*ham*_.

Fig. 7 shows a search scheme with two allowed errors and split into four parts. The node count is identical to the known optimum search schemes.

#### Step by step example

The following section will go through an example creating $\mathcal {S}_{H, 3, 5}$, which, in this case, is an optimum search scheme. It allows searches for up to *k* = 3 errors and splits a query into *p* = *k* + 2 = 5 parts. We start by generating the order of pieces for each search:


\begin{eqnarray*} \pi _0 &= (0, 1, 2, 3, 4)\\ \pi _1 &= (1, 2, 3, 4, 0)\\ \pi _2 &= (2, 3, 4, 1, 0)\\ \pi _3 &= (3, 4, 2, 1, 0)\\ \end{eqnarray*}

Next, we generate the lower bound *L* of every search:


\begin{eqnarray*} L_0 = (0, 0, 0, 0, 3)\\ L_1 = (0, 0, 0, 2, 2)\\ L_2 = (0, 0, 1, 1, 1)\\ L_3 = (0, 0, 0, 0, 0)\\ \end{eqnarray*}

To generate the upper bound, we first need to compute *M*_*ord*_ first. For this, we create an initial matrix *M*_0_:


\begin{eqnarray*} M_0 &= \begin{pmatrix}m_{0,0}&m_{0,1}&m_{0,2}&m_{0,3}&m_{0,4}\\ m_{1,0}&m_{1,1}&m_{1,2}&m_{1,3}&m_{1,4}\\ m_{2,0}&m_{2,1}&m_{2,2}&m_{2,3}&m_{2,4}\\ m_{3,0}&m_{3,1}&m_{3,2}&m_{3,3}&m_{3,4}\\ m_{4,0}&m_{4,1}&m_{4,2}&m_{4,3}&m_{4,4}\\ \end{pmatrix}\\ &= \begin{pmatrix}0&2&1&3&3\\ 1&0&2&2&2\\ 2&1&0&1&1\\ 3&3&3&0&0 \end{pmatrix} \end{eqnarray*}

The value *m*_0, 1_ = 2 and *m*_0, 2_ = 1 violates the property *m*_0, 1_ ≤ *m*_0, 2_. Triggering a permutation of the column 2 results in a new matrix *M*_1_:


\begin{eqnarray*} M_1 &= \begin{pmatrix}0&2&2&3&3\\ 1&0&1&2&2\\ 2&1&0&1&1\\ 3&3&3&0&0 \end{pmatrix} \end{eqnarray*}

This matrix *M*_1_ fulfills all required properties, so we can continue reordering the values according to π to receive *M*_*ord*_:


\begin{eqnarray*} M_{ord} = \begin{pmatrix}0&2&2&3&3\\ 0&1&2&2&1\\ 0&1&1&1&2\\ 0&0&3&3&3 \end{pmatrix} \end{eqnarray*}

To compute *U* we need to combine *L* and *M*_*ord*_.:


\begin{eqnarray*} U_0& = &(\min (0 + 0, k), \min (0 + 2, k), \min (0 + 2, k),\\ && \min (0 + 3, k), \min (3 + 3, k))\\ &= &(0, 2, 2, 3, 3)\\ U_1 &= &(\min (0 + 0, k), \min (0 + 1, k), \min (0 + 2, k),\\ && \min (0 + 2, k), \min (2 + 1, k))\\ &= &(0, 1, 2, 2, 3)\\ U_2 &= &(\min (0 + 0, k), \min (0 + 1, k), \min (0 + 1, k),\\ && \min (1 + 1, k), \min (1 + 2, k))\\ &= &(0, 1, 1, 2, 3)\\ U_3 &= &(\min (0 + 0, k), \min (0 + 0, k), \min (0 + 3, k),\\ && \min (0 + 3, k), \min (0 + 3, k))\\ &= &(0, 0, 3, 3, 3) \end{eqnarray*}

This results in four searches *s*_0_ = (01234, 00003, 02233), *s*_1_ = (12340, 00022, 01223), *s*_2_ = (23410, 00111, 01123), and *s*_3_ = (34210, 00000, 00333).

## Results and discussion

### Evaluation

In this section, we want to evaluate the node count, weighted node count and run time of all search schemes. In our benchmarks, we use a string generated over the alphabet Σ = {*A*, *C*, *G*, *T*} of length 3 billion as our reference text. (Roughly, the size of the human genome). As queries, we created a fixed set of simulated strings of length 50 with an exact number of edit distance errors depending on the benchmark. The length of 50 mimics methods that use the FM-Index as a seeding strategy [16, 17]. For run time measurements, we used the *Columba* tool [13] in version *v1.2*. We activate uniform partitioning and did not use in-text verification to avoid query specific optimization, but we used Columbas “editopt” search distance knowing this will lead to overestimation of our weighted node count. To generate simulated genomes, simulated reads and search schemes, we use our tool *Sahara* [18]. We also use it to simplify the preprocessing steps for *Columba*. While *Sahara* is capable of executing searches, we decided to use the superior implementation in *Columba*, which also maintains the comparability with previous published papers by Renders *et al.*

Using the node count as a performance predictor (Table 1), we expect that $\mathcal {S}_{H, k, k+2}$ and $\mathcal {S}_{OSS, k}$ outperform all other search strategies. Interestingly, it even predicts that $\mathcal {S}_{H, k, k+2}$ outperforms the optimum search scheme $\mathcal {S}_{OSS, k}$ for two errors. We attribute this to the partitioning of the query. Since the query is 50 bases long and divided into four parts, the parts will have either 12 or 13 bases. Depending on the partition, the node count prefers one or the other search scheme.

**Table 1.** Showing the node count of different search schemes. Assuming |*P*| = 50, |Σ| = 4 and edit distance. The lowest node counts are highlighted. Empty cells denote non-existing search schemes

errors	1	2	3	4	5	6
$\mathcal {S}_{bt, k}$	1 025 · 10^1^	1 343 · 10^3^	1 293 · 10^5^	975 · 10^7^	600 · 10^9^	309 · 10^11^
$\mathcal {S}_{OSS, k}$	**530** · 10^1^	**747** · 10^3^	**732** · 10^5^	-	-	-
$\mathcal {S}_{01*0, k}$	585 · 10^1^	836 · 10^3^	1 004 · 10^5^	949 · 10^7^	703 · 10^9^	424 · 10^11^
$\mathcal {S}_{01*0-opt, k}$	581 · 10^1^	1 014 · 10^3^	1 356 · 10^5^	1 279 · 10^7^	951 · 10^9^	567 · 10^11^
$\mathcal {S}_{ph, k}$	**530** · 10^1^	1 199 · 10^3^	1 621 · 10^5^	1 561 · 10^7^	1 166 · 10^9^	703 · 10^11^
$\mathcal {S}_{ph-opt, k}$	**530** · 10^1^	900 · 10^3^	1 062 · 10^5^	938 · 10^7^	649 · 10^9^	368 · 10^11^
$\mathcal {S}_{sf, k}$	**530** · 10^1^	780 · 10^3^	889 · 10^5^	753 · 10^7^	557 · 10^9^	315 · 10^11^
$\mathcal {S}_{H2, k, k+1}$	**530** · 10^1^	779 · 10^3^	**790** · 10^5^	603 · 10^7^	389 · 10^9^	205 · 10^11^
$\mathcal {S}_{H2, k, k+2}$	581 · 10^1^	**737** · 10^3^	**732** · 10^5^	**568** · 10^7^	**359** · 10^9^	**189** · 10^11^
$\mathcal {S}_{H2, k, k+3}$	645 · 10^1^	790 · 10^3^	**753** · 10^5^	**579** · 10^7^	**362** · 10^9^	**189** · 10^11^
$\mathcal {S}_{kianfar, k}$	**530** · 10^1^	779 · 10^3^	1 194 · 10^5^	-	-	-
$\mathcal {S}_{kuch, k, k+1}$	**530** · 10^1^	807 · 10^3^	983 · 10^5^	1 122 · 10^7^	-	-
$\mathcal {S}_{kuch, k, k+2}$	581 · 10^1^	857 · 10^3^	804 · 10^5^	1 055 · 10^7^	-	-
$\mathcal {S}_{hato, k}$	**530** · 10^1^	762 · 10^3^	834 · 10^5^	659 · 10^7^	483 · 10^9^	235 · 10^11^

When measuring the run times for 100 000 queries using the different queries Table 2 tells a different story. Despite our prediction derived from the node count, in every error category our heuristic search schemes $\mathcal {S}_{H, k, k+c}$ and $\mathcal {S}_{OSS, k}$ were outperformed by other schemes. A similar observation was also made by Renders *et al.* [13]. Moreover, our node count prediction indicates that all search schemes will have similar magnitude in run time, especially $\mathcal {S}_{bt, k}$ should not be slower than 2× the fastest search schemes. Contrary to our prediction the actual run times in Table 2 show that $\mathcal {S}_{bt, k}$ is multiple magnitudes slower than every other search schemes. Lastly, from the node count, one should estimate that every additional error increases run time about 100×, predicting run time of $\mathcal {S}_{01*0,k}$ suggests an increase of 10^10^× when changing *k* = 1 to *k* = 6. Our benchmarks show it is less than 10^3^× slower. This is observable for all search schemes. From these observations we conclude that node count is not a useful predictor to estimate the performance of a search scheme.

**Table 2.** Showing the run time in seconds of search schemes run by Columba searching for 100 000 queries. Using |*P*| = 50, |Σ| = 4, |*T*| = 3 000 000 000, with simulated reads using edit distance. The lowest run times are highlighted. For *k* = 5 and *k* = 6, only 1000 queries were executed and the run time scaled by 100. Empty cells denote non-existing search schemes. The search scheme $\mathcal {S}_{H, k, k+3}$ for *k* = 6 failed due to a Columba fallback to a *normal bidirectional search* and $\mathcal {S}_{bt, k}$ timed-out after 1 h

Errors	1	2	3	4	5	6
$\mathcal {S}_{bt, k}$	28.05	725.68	timeout	timeout	timeout	timeout
$\mathcal {S}_{OSS, k}$	**1.54**	5.17	48.13	-	-	-
$\mathcal {S}_{01*0, k}$	1.80	5.92	21.54	70.81	263.00	1084.00
$\mathcal {S}_{01*0-opt, k}$	1.80	5.15	16.44	**58.08**	725.00	9432.00
$\mathcal {S}_{ph, k}$	**1.52**	**2.16**	54.60	1672.09	29 789.00	239 756.00
$\mathcal {S}_{ph-opt, k}$	**1.52**	**2.07**	38.11	860.12	14 862.00	12 0531.00
$\mathcal {S}_{sf, k}$	**1.54**	**2.02**	13.80	357.49	2225.00	12 194.00
$\mathcal {S}_{H2, k, k+1}$	**1.53**	**2.01**	20.41	534.57	5937.00	44 025.00
$\mathcal {S}_{H2, k, k+2}$	1.78	5.04	48.50	928.63	9784.00	78 642.00
$\mathcal {S}_{H2, k, k+3}$	3.75	11.28	145.14	1885.38	19 851.00	failed
$\mathcal {S}_{kianfar, k}$	**1.52**	**2.01**	107.31	-	-	-
$\mathcal {S}_{kuch, k, k+1}$	**1.53**	**2.06**	**8.40**	62.47	-	-
$\mathcal {S}_{kuch, k, k+2}$	1.78	5.23	16.19	**50.56**	-	-
$\mathcal {S}_{hato, k}$	**1.55**	**2.05**	**8.36**	**57.93**	**68.00**	**426.00**

As an alternative run time estimation, we compare our previously introduced weighted node count with the run time. Table 3 lists the different weighted node counts. Here we see correlation between the predicted fastest search schemes and the actual run time. Only for *k* = 4, our prediction of fastest and second-fastest search scheme is swapped. The weighted node count correctly predicts that $\mathcal {S}_{bt,k}$ is magnitudes slower than the other search schemes.

**Table 3.** Showing the weighted node count of different search schemes. Assuming |*P*| = 50, |Σ| = 4, |*T*| = 3 000 000 000 and edit distance. The lowest weighted node counts are highlighted. Empty cells denote non-existing search schemes

Errors	1	2	3	4	5	6
$\mathcal {S}_{bt, k}$	1 082.60	434 . 10^2^	122 . 10^4^	255 . 10^5^	414 . 10^6^	530 . 10^9^
$\mathcal {S}_{OSS, k}$	**31.65**	198.46	2 669.57	-	-	-
$\mathcal {S}_{01*0, k}$	47.52	229.60	1 122.48	2 650.15	15 086.29	123 820.56
$\mathcal {S}_{01*0-opt, k}$	**31.70**	137.24	545.41	**1 255.75**	46 111.73	2 879 754.09
$\mathcal {S}_{ph, k}$	**31.65**	**48.99**	8 531.56	530 041.98	15 683 373.87	253 620 952.60
$\mathcal {S}_{ph-opt, k}$	**31.65**	**48.63**	2 678.92	233 968.19	3 128 519.06	94 408 630.87
$\mathcal {S}_{sf, k}$	**31.65**	**48.98**	3 534.11	106 714.51	3 313 394.15	40 034 994.27
$\mathcal {S}_{H2, k, k+1}$	**31.65**	**48.98**	3 782.52	128 136.32	3 617 506.01	44 548 566.52
$\mathcal {S}_{H2, k, k+2}$	**31.70**	137.24	2 669.57	42 121.71	684 323.68	9 127 067.20
$\mathcal {S}_{H2, k, k+3}$	76.53	368.88	4 184.41	76 653.18	1 224 984.33	18 787 288.71
$\mathcal {S}_{kianfar, k}$	**31.65**	**48.98**	6 323.76	-	-	-
$\mathcal {S}_{kuch, k, k+1}$	**31.65**	**48.63**	**304.05**	3 375.69	-	-
$\mathcal {S}_{kuch, k, k+2}$	**31.70**	183.68	545.41	**1 497.72**	-	-
$\mathcal {S}_{hato, k}$	**31.65**	**48.63**	**304.05**	3 006.80	**1 996.37**	**31 105.20**

If we compare across error categories, e.g. $\mathcal {S}_{01*0,k}$, we predict an increase of 2600 × going from *k* = 1 to *k* = 6. In our measured data, we see an actual increase of 600×. Similar correlation can be made across all search schemes. We are assuming the deviations originate from the optimizations of the Columba tool, which only follows a reduced set of edges. We normalized the (weighted) node count by a factor, such that $\mathcal {S}_{OSS,k}$ for *k* = 1 is perfectly predicted. (We have chosen $\mathcal {S}_{OSS,k}$ for no specific reason. The tables look similar if a different search scheme is chosen for normalization.) The value is then divided by the actual run time, giving us a ratio. The resulting numbers are shown in Supplementary Tables S1 and S2. The weighted node count is often a very good predictor of the run time (mostly within a factor of 2). The maximal overestimation is in one case 160× while the node count over-estimates 16 · 10^6^×.

The weighted node count is not a perfect predictor of a search scheme performance, but it is a superior estimator compared to the node count approach. It allows to identify a well performing search scheme considering the number of allowed errors, the query length and the length of the reference text.

We ran the same benchmark on a fixed set of simulated strings of length 150, which is a typical size of reads produced by Illumina sequencing machines. The results are less informative and can be found in the Supplementary Tables S3–S5. The run-times within an error category are close such that expressive derivations are harder to show, and we decided to focus on reads of length 50 instead. Additionally, we ran the benchmark using the human genome with a read length of 50 and 150. The results can be seen in Supplementary Tables S6 and S7. While these results look similar, they require the weighted node count to take the self-similarity of the reference genome into account. We decided to adhere to the random generated reference, because it is a simpler model.

In summary, we see that $\mathcal {S}_{hato,k}$ has the lowest run time (with exception of *k* = 4) even though it does not have the lowest node count. Our weighted node count prediction is closer to the real run time compared to the node count. The node count wrongly predicts that $\mathcal {S}_{H, k, k+2}$ is the fastest for all *k* ≥ 2. In contrast, the weighted node count predicts the fastest search scheme for all error category correctly, missing the real run-time by a small factor.

## Conclusion

We formulated different search strategies as search schemes and introduced our own search scheme construction algorithm. This enables the creation of search schemes with any number of errors. We showed that our search scheme achieves the lowest node count in every error category, but run times expose that there is no correlation between the run time and the node count. We used this knowledge to introduce an improved weighted node count, which can be used to estimate run times to a certain degree of certainty. This allows to choose the best search scheme for a given set of queries, which confirms the finding of Renders *et al.* [14]. Although, there is space to explore even better predictors, the weighted node count can be used for future development of new search scheme constructions that enable better performance.

## Supplementary Material

lqaf025_Supplemental_File

## Data Availability

Data are available at https://github.com/seqan/sahara (https://doi.org/10.5281/zenodo.14640235) and https://github.com/SGSSGene/sahara_benchmarks (https://doi.org/10.5281/zenodo.14640239).

## Appendix

Weighted node count

\begin{eqnarray*} &n_{edit}(\mathcal {S}) = \sum _{(\pi , \mathcal {L}, \mathcal {U}) \in \mathcal {S}} n_{edit}(\pi , \mathcal {L}, \mathcal {U})\\ &n_{edit}(\pi , \mathcal {L}, \mathcal {U}) = \sum _{d=\mathcal {L}_{|\pi |-1}}^{\mathcal {U}_{|\pi |-1}} n_{|\pi |-1, d}\\ &n_{l, d} = \left\lbrace \begin{array}{@{}l@{\quad }l@{}}(1) & \mathcal {L}_l \le d \le \mathcal {U}_l \wedge 1 \le l \le \lceil \log _{\sigma } |T| \rceil \\ (2) & \mathcal {L}_l \le d \le \mathcal {U}_l \wedge l > \lceil \log _{\sigma } |T| \rceil \\ 1 & l = 0, d = 0 \\ 0 & otherwise \end{array}\right.\\ &(1) = n_{l-1, d} + (\sigma -1) \cdot n_{l-1, d-1} + \sigma \cdot n_{l, d-1} + n_{l-1, d-1}\\ &(2) = (n_{l-1, d} + (\sigma -1) \cdot n_{l-1, d-1} + \sigma \cdot n_{l, d-1} + n_{l-1, d-1}) \cdot \frac{|T|}{\sigma ^{l}} \end{eqnarray*}

Weighted node count for edit distance. In most practical application, this formula results in an overestimation, because some recursive calls can be optimized.
