# Supplementary material for: SeArcH schemes for Approximate stRing mAtching
Source: NAR Genom Bioinform. 2025 Mar 18;7(1):lqaf025. doi: 10.1093/nargab/lqaf025 (PMC11915513; doi:10.1093/nargab/lqaf025)
Supplement: lqaf025_Supplemental_File [file lqaf025_supplemental_file.pdf]

Supplementary

SeArcH schemes for Approximate stRing mAtching

Simon G. Gottlieb & Knut Reinert

1 Ratio of runtime and (weighted) node count

errors	1	2	3	4	5	6
$\mathcal{S}_{bt,k}$	0.10618	0.53774	-	-	-	-
$\mathcal{S}_{OSS,k}$	1	41.983	441.92	-	-	-
$\mathcal{S}_{01*0,k}$	0.94434	41.033	1354.4	38942	7.7668e+05	1.1365e+07
$\mathcal{S}_{01*0-opt,k}$	0.93788	57.21	2396.6	63987	3.8114e+05	1.7467e+06
$\mathcal{S}_{ph,k}$	1.0132	161.29	862.65	2712.6	11373	85198
$\mathcal{S}_{ph-opt,k}$	1.0132	126.33	809.71	3168.8	12689	88714
$\mathcal{S}_{sf,k}$	1	112.2	1871.8	6120.3	72739	7.506e+05
$\mathcal{S}_{H2,k,k+1}$	1.0065	112.61	1124.7	3277.6	19038	1.353e+05
$\mathcal{S}_{H2,k,k+2}$	0.94842	42.49	438.55	1777.3	10662	69832
$\mathcal{S}_{H2,k,k+3}$	0.49977	20.35	150.75	892.33	5298.7	-
$\mathcal{S}_{kianfar,k}$	1.0132	112.61	323.3	-	-	-
$\mathcal{S}_{kuch,k,k+1}$	1.0065	113.83	3400.3	52187	-	-
$\mathcal{S}_{kuch,k,k+2}$	0.94842	47.613	1443	60630	-	-
$\mathcal{S}_{hato,k}$	0.99355	108.01	2898.7	33054	2.0639e+06	1.6029e+07

Table Supplementary Table S1: Showing ratio of Table 1 and Table 2 (see main article) normalized for the first entry of $\mathcal{S}_{OSS,k}$, $k = 1$. A value of 1 denotes a node count predicted the run-time perfectly. A value of 2 means that the predictor over-estimated the run-time by 2. A value of 0.5 means the predictor under-estimated the run-time by 0.5

errors	1	2	3	4	5	6
$\mathcal{S}_{bt,k}$	1.8779	2.91	-	-	-	-
$\mathcal{S}_{OSS,k}$	1	1.8678	2.6988	-	-	-
$\mathcal{S}_{01*0,k}$	1.2845	1.8871	2.5356	1.8211	2.7911	5.5579
$\mathcal{S}_{01*0-opt,k}$	0.85691	1.2966	1.6142	1.052	3.0947	14.856
$\mathcal{S}_{ph,k}$	1.0132	1.1036	7.603	15.424	25.617	51.471
$\mathcal{S}_{ph-opt,k}$	1.0132	1.1431	3.4203	13.236	10.243	38.112
$\mathcal{S}_{sf,k}$	1	1.1798	12.461	14.525	72.459	159.75
$\mathcal{S}_{H2,k,k+1}$	1.0065	1.1857	9.0175	11.663	29.648	49.236
$\mathcal{S}_{H2,k,k+2}$	0.86654	1.3249	2.6782	2.207	3.4032	5.6471
$\mathcal{S}_{H2,k,k+3}$	0.993	1.5912	1.4028	1.9782	3.0026	-
$\mathcal{S}_{kianfar,k}$	1.0132	1.1857	2.8674	-	-	-
$\mathcal{S}_{kuch,k,k+1}$	1.0065	1.1486	1.7612	2.6293	-	-
$\mathcal{S}_{kuch,k,k+2}$	0.86654	1.7089	1.6392	1.4414	-	-
$\mathcal{S}_{hato,k}$	0.99355	1.1542	1.7696	2.5255	1.4285	3.5528

Table Supplementary Table S2: Showing ratio of Table 3 and Table 2 (see main article) normalized for the first entry of $\mathcal{S}_{OSS,k}$, $k = 1$. A value of 1 denotes a node count predicted the run-time perfectly. A value of 2 means that the predictor over-estimated the run-time by 2. A value of 0.5 means the predictor under-estimated the run-time by 0.5

2 Benchmark for reads of length 150

errors	1	2	3	4	5	6
$\mathcal{S}_{bt,k}$	$907 \cdot 10^2$	$361 \cdot 10^5$	$1069 \cdot 10^7$	$252 \cdot 10^{10}$	$490 \cdot 10^{12}$	$813 \cdot 10^{14}$
$\mathcal{S}_{OSS,k}$	$\mathbf{459 \cdot 10^2}$	$\mathbf{195 \cdot 10^5}$	$\mathbf{586 \cdot 10^7}$	–	–	–
$\mathcal{S}_{01*0,k}$	$510 \cdot 10^2$	$221 \cdot 10^5$	$824 \cdot 10^7$	$243 \cdot 10^{10}$	$575 \cdot 10^{12}$	$1113 \cdot 10^{14}$
$\mathcal{S}_{01*0-opt,k}$	$509 \cdot 10^2$	$271 \cdot 10^5$	$1113 \cdot 10^7$	$331 \cdot 10^{10}$	$776 \cdot 10^{12}$	$1495 \cdot 10^{14}$
$\mathcal{S}_{ph,k}$	$\mathbf{459 \cdot 10^2}$	$321 \cdot 10^5$	$1349 \cdot 10^7$	$409 \cdot 10^{10}$	$974 \cdot 10^{12}$	$1907 \cdot 10^{14}$
$\mathcal{S}_{ph-opt,k}$	$\mathbf{459 \cdot 10^2}$	$242 \cdot 10^5$	$877 \cdot 10^7$	$242 \cdot 10^{10}$	$533 \cdot 10^{12}$	$979 \cdot 10^{14}$
$\mathcal{S}_{sf,k}$	$\mathbf{459 \cdot 10^2}$	$203 \cdot 10^5$	$707 \cdot 10^7$	$190 \cdot 10^{10}$	$420 \cdot 10^{12}$	$800 \cdot 10^{14}$
$\mathcal{S}_{H2,k,k+1}$	$\mathbf{459 \cdot 10^2}$	$203 \cdot 10^5$	$630 \cdot 10^7$	$151 \cdot 10^{10}$	$299 \cdot 10^{12}$	$506 \cdot 10^{14}$
$\mathcal{S}_{H2,k,k+2}$	$509 \cdot 10^2$	$\mathbf{194 \cdot 10^5}$	$\mathbf{586 \cdot 10^7}$	$\mathbf{140 \cdot 10^{10}}$	$\mathbf{277 \cdot 10^{12}}$	$\mathbf{464 \cdot 10^{14}}$
$\mathcal{S}_{H2,k,k+3}$	$569 \cdot 10^2$	$207 \cdot 10^5$	$613 \cdot 10^7$	$\mathbf{144 \cdot 10^{10}}$	$\mathbf{284 \cdot 10^{12}}$	$\mathbf{474 \cdot 10^{14}}$
$\mathcal{S}_{kianfar,k}$	$\mathbf{459 \cdot 10^2}$	$203 \cdot 10^5$	$984 \cdot 10^7$	–	–	–
$\mathcal{S}_{kuch,k,k+1}$	$\mathbf{459 \cdot 10^2}$	$216 \cdot 10^5$	$799 \cdot 10^7$	$285 \cdot 10^{10}$	–	–
$\mathcal{S}_{kuch,k,k+2}$	$509 \cdot 10^2$	$223 \cdot 10^5$	$647 \cdot 10^7$	$262 \cdot 10^{10}$	–	–
$\mathcal{S}_{hato,k}$	$\mathbf{459 \cdot 10^2}$	$203 \cdot 10^5$	$675 \cdot 10^7$	$167 \cdot 10^{10}$	$393 \cdot 10^{12}$	$605 \cdot 10^{14}$

Table Supplementary Table S3: Showing the node count of different search schemes. Assuming $|P| = 150$, $|\Sigma| = 4$ and edit distance. The lowest node counts are highlighted. Empty cells denote non-existing search schemes.

errors	1	2	3	4	5	6
$\mathcal{S}_{bt,k}$	1 082.60	43401.44	1220232.67	25575336.98	414206480.90	5301485727.89
$\mathcal{S}_{OSS,k}$	**31.65**	**47.48**	**63.30**	-	-	-
$\mathcal{S}_{01*0,k}$	47.48	94.96	158.26	237.39	332.34	443.12
$\mathcal{S}_{01*0-opt,k}$	**31.65**	**47.48**	**63.30**	**79.13**	**94.96**	**110.78**
$\mathcal{S}_{ph,k}$	**31.65**	**47.48**	**63.30**	**79.13**	**94.96**	**110.78**
$\mathcal{S}_{ph-opt,k}$	**31.65**	**47.48**	**63.30**	**79.13**	**94.96**	**110.78**
$\mathcal{S}_{sf,k}$	**31.65**	**47.48**	**63.30**	**79.13**	**94.96**	**110.78**
$\mathcal{S}_{H2,k,k+1}$	**31.65**	**47.48**	**63.30**	**79.13**	**94.96**	**110.78**
$\mathcal{S}_{H2,k,k+2}$	**31.65**	**47.48**	**63.30**	**79.13**	**94.96**	**110.78**
$\mathcal{S}_{H2,k,k+3}$	**31.65**	**47.48**	**63.30**	**79.13**	**94.96**	**111.06**
$\mathcal{S}_{kianfar,k}$	**31.65**	**47.48**	1114.25	-	-	-
$\mathcal{S}_{kuch,k,k+1}$	**31.65**	**47.48**	**63.30**	126.61	-	-
$\mathcal{S}_{kuch,k,k+2}$	**31.65**	63.30	**63.30**	158.26	-	-
$\mathcal{S}_{hato,k}$	**31.65**	**47.48**	**63.30**	**79.13**	**94.96**	**110.78**

Table Supplementary Table S4: Showing the weighted node count of different search schemes. Assuming $|P| = 150$, $|\Sigma| = 4$, $|T| = 3\,000\,000\,000$ and edit distance. The lowest weighted node counts are highlighted. Empty cells denote non-existing search schemes.

errors	1	2	3	4	5	6
$\mathcal{S}_{bt,k}$	30.69	726.25	timeout	timeout	timeout	timeout
$\mathcal{S}_{OSS,k}$	**3.41**	4.46	**4.72**	-	-	-
$\mathcal{S}_{01*0,k}$	**3.62**	4.44	5.20	6.09	6.99	8.03
$\mathcal{S}_{01*0-opt,k}$	**3.59**	**4.27**	4.98	5.67	6.40	7.18
$\mathcal{S}_{ph,k}$	**3.43**	**4.22**	5.01	5.82	6.75	7.58
$\mathcal{S}_{ph-opt,k}$	**3.41**	**4.01**	**4.67**	**5.15**	**5.73**	**6.26**
$\mathcal{S}_{sf,k}$	**3.43**	**3.98**	**4.52**	**5.13**	**5.65**	**6.16**
$\mathcal{S}_{H2,k,k+1}$	**3.40**	**3.98**	**4.58**	**5.15**	**5.69**	**6.25**
$\mathcal{S}_{H2,k,k+2}$	**3.60**	**4.09**	**4.59**	**5.14**	**5.76**	**6.35**
$\mathcal{S}_{H2,k,k+3}$	3.85	**4.34**	**4.85**	**5.37**	**6.04**	7.12
$\mathcal{S}_{kianfar,k}$	**3.41**	**3.98**	34.56	-	-	-
$\mathcal{S}_{kuch,k,k+1}$	**3.41**	**4.01**	**4.57**	5.73	-	-
$\mathcal{S}_{kuch,k,k+2}$	**3.57**	4.56	**4.66**	6.17	-	-
$\mathcal{S}_{hato,k}$	**3.43**	**3.98**	**4.40**	**4.97**	**5.46**	**5.79**

Table Supplementary Table S5: Showing the run time in seconds of search schemes run by Columba searching for 100 000 queries. Using $|P| = 150$, $|\Sigma| = 4$, $|T| = 3\ 000\ 000\ 000$, with simulated reads using edit distance. The lowest run times are highlighted. The search scheme $\mathcal{S}_{bt,k}$ timed-out after one hour.

3 Benchmark for human genome for reads of length 50 and 150

errors	1	2	3	4	5	6
$\mathcal{S}_{bt,k}$	41.50s	763.58s	timeout	timeout	timeout	timeout
$\mathcal{S}_{OSS,k}$	9.93s	22.25s	93.40s	-	-	-
$\mathcal{S}_{01*0,k}$	11.31s	24.38s	67.11s	216.27s	686.00s	2755.00s
$\mathcal{S}_{01*0-opt,k}$	11.09s	24.11s	77.92s	328.51s	2061.00s	17110.00s
$\mathcal{S}_{ph,k}$	9.92s	26.80s	203.60s	2693.06s	35583.00s	258991.00s
$\mathcal{S}_{ph-opt,k}$	9.78s	23.12s	133.35s	1393.01s	17033.00s	125481.00s
$\mathcal{S}_{sf,k}$	9.87s	19.53s	67.33s	628.91s	3054.00s	16114.00s
$\mathcal{S}_{H,k,k+1}$	9.86s	19.44s	80.03s	853.52s	7000.00s	45079.00s
$\mathcal{S}_{H,k,k+2}$	11.00s	22.03s	92.23s	1059.28s	9340.00s	68845.00s
$\mathcal{S}_{H,k,k+3}$	13.96s	32.14s	197.20s	1924.22s	17711.00s	failed
$\mathcal{S}_{kianfar,k}$	9.89s	19.28s	179.94s	-	-	-
$\mathcal{S}_{kuch,k,k+1}$	9.89s	20.30s	42.64s	157.34s	-	-
$\mathcal{S}_{kuch,k,k+2}$	11.01s	22.61s	51.62s	148.09s	-	-
$\mathcal{S}_{hato,k}$	9.87s	20.49s	41.15s	142.46s	238.00s	803.00s

Table Supplementary Table S6: Showing the run time in seconds of search schemes run by Columba searching for 100 000 queries. Using $|P| = 50$, $|\Sigma| = 4$, $T =$ human genome, with simulated reads using edit distance. The lowest run times are highlighted. For $k = 5$ and $k = 6$ only 1 000 queries were executed and the run time scaled by 100. Empty cells denote non-existing search schemes. The search scheme $\mathcal{S}_{H,k,k+3}$ for $k = 6$ failed due to a Columba fallback to a *normal bidirectional search* and $\mathcal{S}_{bt,k}$ timed-out after one hour.

errors	1	2	3	4	5	6
$\mathcal{S}_{bt,k}$	39.87s	797.34s	timeout	timeout	timeout	timeout
$\mathcal{S}_{OSS,k}$	4.09s	6.25s	9.64s	-	-	-
$\mathcal{S}_{01*0,k}$	4.51s	6.41s	9.53s	15.43s	25.52s	45.13s
$\mathcal{S}_{01*0-opt,k}$	4.52s	6.23s	9.48s	16.70s	33.51s	75.05s
$\mathcal{S}_{ph,k}$	4.06s	6.47s	14.33s	43.42s	131.84s	373.03s
$\mathcal{S}_{ph-opt,k}$	4.08s	5.92s	11.18s	27.77s	73.99s	202.65s
$\mathcal{S}_{sf,k}$	4.08s	5.55s	8.55s	15.98s	32.89s	65.89s
$\mathcal{S}_{H,k,k+1}$	4.08s	5.50s	9.24s	19.92s	50.89s	129.50s
$\mathcal{S}_{H,k,k+2}$	4.54s	5.86s	9.41s	19.61s	50.43s	131.49s
$\mathcal{S}_{H,k,k+3}$	5.18s	7.11s	12.49s	30.08s	78.18s	191.67s
$\mathcal{S}_{kianfar,k}$	4.08s	5.58s	47.67s	-	-	-
$\mathcal{S}_{kuch,k,k+1}$	4.09s	5.59s	7.18s	12.22s	-	-
$\mathcal{S}_{kuch,k,k+2}$	4.54s	6.39s	8.03s	13.21s	-	-
$\mathcal{S}_{hato,k}$	4.07s	6.61s	7.03s	10.63s	14.00s	20.51s

Table Supplementary Table S7: Showing the run time in seconds of search schemes run by Columba searching for 100 000 queries. Using $|P| = 150$, $|\Sigma| = 4$, $|T| =$ human genome, with simulated reads using edit distance. The lowest run times are highlighted. The search scheme $\mathcal{S}_{bt,k}$ timed-out after one hour.

4 Search Schemes

$\mathcal{S}_{bt,k}$

- $k = 1$: $\{(0, 0, 1)\}$
- $k = 2$: $\{(0, 0, 2)\}$

- $k = 3$: $\{(0, 0, 3)\}$
- $k = 4$: $\{(0, 0, 4)\}$
- $k = 5$: $\{(0, 0, 5)\}$
- $k = 6$: $\{(0, 0, 6)\}$

$\mathcal{S}_{OSS,k}$

- $k = 1$: $\{(01, 00, 01), (10, 01, 01)\}$
- $k = 2$: $\{(0123, 0011, 0022), (2103, 0000, 0112), (3210, 0002, 0122)\}$
- $k = 3$: $\{(43210, 00000, 00333), (23410, 00111, 01123), (12340, 00022, 01223), (01234, 00003, 02233)\}$

$\mathcal{S}_{01*0,k}$

- $k = 1$: $\{(012, 000, 001), (012, 011, 011), (120, 000, 001)\}$
- $k = 2$: $\{(0123, 0000, 0022), (0123, 0111, 0112), (0123, 0122, 0122), (1230, 0000, 0022), (1230, 0111, 0112), (2310, 0000, 0022)\}$
- $k = 3$: $\{(01234, 00000, 00333), (01234, 01111, 01133), (01234, 01222, 01223), (01234, 01233, 01233), (12340, 00000, 00333), (12340, 01111, 01133), (12340, 01222, 01223), (23410, 00000, 00333), (23410, 01111, 01133), (34210, 00000, 00333)\}$
- $k = 4$: $\{(012345, 000000, 004444), (012345, 011111, 011444), (012345, 012222, 012244), (012345, 012333, 012334), (012345, 012344, 012344), (123450, 000000, 004444), (123450, 011111, 011444), (123450, 012222, 012244), (123450, 012333, 012334), (234510, 000000, 004444), (234510, 011111, 011444), (234510, 012222, 012244), (345210, 000000, 004444), (345210, 011111, 011444), (453210, 000000, 004444)\}$
- $k = 5$: $\{(0123456, 0000000, 0055555), (0123456, 0111111, 0115555), (0123456, 0122222, 0122555), (0123456, 0123333, 0123355), (0123456, 0123444, 0123445), (0123456, 0123455, 0123455), (1234560, 0000000, 0055555), (1234560, 0111111, 0115555), (1234560, 0122222, 0122555), (1234560, 0123333, 0123355), (1234560, 0123444, 0123445), (2345610, 0000000, 0055555), (2345610, 0111111, 0115555), (2345610, 0122222, 0122555), (2345610, 0123333, 0123355), (3456210, 0000000, 0055555), (3456210, 0111111, 0115555), (3456210, 0122222, 0122555), (4563210, 0000000, 0055555), (4563210, 0111111, 0115555), (5643210, 0000000, 0055555)\}$
- $k = 6$: $\{(01234567, 00000000, 00666666), (01234567, 01111111, 01166666), (01234567, 01222222, 01226666), (01234567, 01233333, 01233666), (01234567, 01234444, 01234466), (01234567, 01234555, 01234556), (01234567, 01234566, 01234566), (12345670, 00000000, 00666666), (12345670, 01111111, 01166666), (12345670, 01222222, 01226666), (12345670, 01233333, 01233666), (12345670, 01234444, 01234466), (12345670, 01234555, 01234556), (23456710, 00000000, 00666666), (23456710, 01111111, 01166666), (23456710, 01222222, 01226666), (23456710, 01233333, 01233666), (23456710, 01234444, 01234466), (34567210, 00000000, 00666666), (34567210, 01111111, 01166666), (34567210, 01222222, 01226666), (34567210, 01233333, 01233666), (45673210, 00000000, 00666666), (45673210, 01111111, 01166666), (45673210, 01222222, 01226666), (56743210, 00000000, 00666666), (56743210, 01111111, 01166666), (67543210, 00000000, 00666666)\}$

$\mathcal{S}_{01*0-opt,k}$

- $k = 1$: $\{(012, 000, 011), (120, 000, 001)\}$
- $k = 2$: $\{(0123, 0000, 0122), (1230, 0000, 0122), (2310, 0000, 0022)\}$
- $k = 3$: $\{(01234, 00000, 01333),$

$(12340, 00000, 01333)$, $(23410, 00000, 01333)$, $(34210, 00000, 00333)\}$

- $k = 4$: $\{(012345, 000000, 014444)$, $(123450, 000000, 014444)$, $(234510, 000000, 014444)$, $(345210, 000000, 014444)$, $(453210, 000000, 004444)\}$
- $k = 5$: $\{(0123456, 0000000, 0155555)$, $(1234560, 0000000, 0155555)$, $(2345610, 0000000, 0155555)$, $(3456210, 0000000, 0155555)$, $(4563210, 0000000, 0155555)$, $(5643210, 0000000, 0055555)\}$
- $k = 6$: $\{(01234567, 00000000, 01666666)$, $(12345670, 00000000, 01666666)$, $(23456710, 00000000, 01666666)$, $(34567210, 00000000, 01666666)$, $(45673210, 00000000, 01666666)$, $(56743210, 00000000, 01666666)$, $(67543210, 00000000, 00666666)\}$

$\mathcal{S}_{ph,k}$

- $k = 1$: $\{(01, 00, 01)$, $(10, 00, 01)\}$
- $k = 2$: $\{(012, 000, 022)$, $(102, 000, 022)$, $(210, 000, 022)\}$
- $k = 3$: $\{(0123, 0000, 0333)$, $(1023, 0000, 0333)$, $(2103, 0000, 0333)$, $(3210, 0000, 0333)\}$
- $k = 4$: $\{(01234, 00000, 04444)$, $(10234, 00000, 04444)$, $(21034, 00000, 04444)$, $(32104, 00000, 04444)$, $(43210, 00000, 04444)\}$
- $k = 5$: $\{(012345, 000000, 055555)$, $(102345, 000000, 055555)$, $(210345, 000000, 055555)$, $(321045, 000000, 055555)$, $(432105, 000000, 055555)$, $(543210, 000000, 055555)\}$
- $k = 6$: $\{(0123456, 0000000, 0666666)$, $(1023456, 0000000, 0666666)$, $(2103456, 0000000, 0666666)$, $(3210456, 0000000, 0666666)$, $(4321056, 0000000, 0666666)$, $(5432106, 0000000, 0666666)$, $(6543210, 0000000, 0666666)\}$

$\mathcal{S}_{ph-opt,k}$

- $k = 1$: $\{(01, 00, 01)$, $(10, 01, 01)\}$ 0
- $k = 2$: $\{(012, 000, 022)$, $(102, 011, 022)$, $(210, 012, 012)\}$
- $k = 3$: $\{(0123, 0000, 0333)$, $(1023, 0111, 0333)$, $(2103, 0122, 0233)$, $(3210, 0123, 0123)\}$
- $k = 4$: $\{(01234, 00000, 04444)$, $(10234, 01111, 04444)$, $(21034, 01222, 03444)$, $(32104, 01233, 02344)$, $(43210, 01234, 01234)\}$
- $k = 5$: $\{(012345, 000000, 055555)$, $(102345, 011111, 055555)$, $(210345, 012222, 045555)$, $(321045, 012333, 034555)$, $(432105, 012344, 023455)$, $(543210, 012345, 012345)\}$
- $k = 6$: $\{(0123456, 0000000, 0666666)$, $(1023456, 0111111, 0666666)$, $(2103456, 0122222, 0566666)$, $(3210456, 0123333, 0456666)$, $(4321056, 0123444, 0345666)$, $(5432106, 0123455, 0234566)$, $(6543210, 0123456, 0123456)\}$

$\mathcal{S}_{sf,k}$

- $k = 1$: $\{(01, 00, 01)$, $(10, 01, 01)\}$
- $k = 2$: $\{(012, 000, 012)$, $(120, 001, 012)$, $(210, 011, 022)\}$
- $k = 3$: $\{(0123, 0000, 0123)$, $(1230, 0001, 0123)$, $(2310, 0011, 0133)$, $(3210, 0111, 0333)\}$
- $k = 4$: $\{(01234, 00000, 01234)$, $(12340, 00001, 01234)$, $(23410, 00011, 01244)$, $(34210, 00111, 01444)$, $(43210, 01111, 04444)\}$
- $k = 5$: $\{(012345, 000000, 012345)$, $(123450, 000001, 012345)$, $(234510, 000011, 012355)$, $(345210, 000111, 012555)$, $(453210, 001111, 015555)$, $(543210, 011111, 055555)\}$
- $k = 6$: $\{(0123456, 0000000, 0123456)$, $(1234560, 0000001, 0123456)$, $(2345610, 0000011, 0123466)$, $(3456210, 0000111, 0123666)$, $(4563210, 0001111, 0126666)$, $(5643210, 0011111, 0166666)$, $(6543210, 0111111, 0666666)\}$

$\mathcal{S}_{H,k,k+1}$

- $k = 1$: $\{(10, 00, 01),\ (01, 01, 01)\}$
- $k = 2$: $\{(210, 000, 022),\ (120, 011, 012),\ (012, 002, 012)\}$
- $k = 3$: $\{(3210, 0000, 0333),\ (2310, 0111, 0123),\ (1230, 0022, 0123),\ (0123, 0003, 0223)\}$
- $k = 4$: $\{(43210, 00000, 04444),\ (34210, 01111, 01234),\ (23410, 00222, 01234),\ (12340, 00033, 02234),\ (01234, 00004, 03334)\}$
- $k = 5$: $\{(543210, 000000, 055555),\ (453210, 011111, 012345),\ (345210, 002222, 012345),\ (234510, 000333, 022345),\ (123450, 000044, 033345),\ (012345, 000005, 044445)\}$
- $k = 6$: $\{(6543210, 0000000, 0666666),\ (5643210, 0111111, 0123456),\ (4563210, 0022222, 0123456),\ (3456210, 0003333, 0223456),\ (2345610, 0000444, 0333456),\ (1234560, 0000055, 0444456),\ (0123456, 0000006, 0555556)\}$

$\mathcal{S}_{H,k,k+2}$

- $k = 1$: $\{(120, 000, 001),\ (012, 001, 011)\}$
- $k = 2$: $\{(2310, 0000, 0022),\ (1230, 0011, 0112),\ (0123, 0002, 0122)\}$
- $k = 3$: $\{(34210, 00000, 00333),\ (23410, 00111, 01123),\ (12340, 00022, 01223),\ (01234, 00003, 02233)\}$
- $k = 4$: $\{(453210, 000000, 004444),\ (345210, 001111, 011234),\ (234510, 000222, 012234),\ (123450, 000033, 022334),\ (012345, 000004, 033344)\}$
- $k = 5$: $\{(5643210, 0000000, 0055555),\ (4563210, 0011111, 0112345),\ (3456210, 0002222, 0122345),\ (2345610, 0000333, 0223345),\ (1234560, 0000044, 0333445),\ (0123456, 0000005, 0444455)\}$
- $k = 6$: $\{(67543210, 00000000, 00666666),\ (56743210, 00111111, 01123456),\ (45673210, 00022222, 01223456),\ (34567210, 00003333, 02233456),\ (23456710, 00000444, 03334456),\ (12345670, 00000055, 04444556),\ (01234567, 00000006, 05555566)\}$

$\mathcal{S}_{H,k,k+3}$

- $k = 1$: $\{(1230, 0000, 0001),\ (0123, 0001, 0111)\}$
- $k = 2$: $\{(23410, 00000, 00022),\ (12340, 00011, 01112),\ (01234, 00002, 01222)\}$
- $k = 3$: $\{(345210, 000000, 000333),\ (234510, 000111, 011123),\ (123450, 000022, 012223),\ (012345, 000003, 022333)\}$
- $k = 4$: $\{(4563210, 0000000, 0004444),\ (3456210, 0001111, 0111234),\ (2345610, 0000222, 0122234),\ (1234560, 0000033, 0223334),\ (0123456, 0000004, 0333444)\}$
- $k = 5$: $\{(56743210, 00000000, 00055555),\ (45673210, 00011111, 01112345),\ (34567210, 00002222, 01222345),\ (23456710, 00000333, 02233345),\ (12345670, 00000044, 03334445),\ (01234567, 00000005, 04444555)\}$
- $k = 6$: $\{(678543210, 000000000, 000666666),\ (567843210, 000111111, 011123456),\ (456783210, 000022222, 012223456),\ (345678210, 000003333, 022333456),\ (234567810, 000000444, 033344456),\ (123456780, 000000055, 044445556),\ (012345678, 000000006, 055555666)\}$

$\mathcal{S}_{kianfar,k}$

- $k = 1$: $\{(01, 00, 01),\ (10, 01, 01)\}$
- $k = 2$: $\{(012, 002, 012),\ (210, 000, 022),\ (120, 011, 012)\}$
- $k = 3$: $\{(0123, 0003, 0233),\ (1230, 0000, 1233),\ (2310, 0022, 0033)\}$

$\mathcal{S}_{kuch,k,k+1}$

- $k = 1$: $\{(01, 00, 01),\ (10, 00, 01)\}$
- $k = 2$: $\{(012, 000, 022),\ (210, 000, 012),\ (102, 001, 012)\}$
- $k = 3$: $\{(0123, 0000, 0133),\ (1023, 0011, 0133),\ (2310, 0000, 0133),\ (3210, 0011, 0133)\}$
- $k = 4$: $\{(01234, 00000, 02244),\ (43210, 00000, 01344),\ (10234, 00133, 01344),$

$(01234, 00133, 01344)$,
$(32410, 00011, 01244)$,
$(21034, 00013, 01244)$,
$(10234, 00124, 01244)$,
$(01234, 00034, 00444)\}$

$\mathcal{S}_{kuch,k,k+2}$

- $k = 1$: $\{(012, 000, 011),\ (120, 000, 001)\}$
- $k = 2$: $\{(0123, 0000, 0112),$ $(3210, 0000, 0122),$ $(1230, 0001, 0012),$ $(0123, 0002, 0022)\}$
- $k = 3$: $\{(01234, 00000, 01233),$ $(12340, 00000, 01223),$ $(23410, 00001, 01133),$ $(34210, 00012, 00333)\}$
- $k = 4$: $\{(012345, 000000, 012344),$ $(123450, 000000, 012344),$ $(543210, 000001, 012244),$ $(345210, 000012, 011344),$ $(234510, 000023, 011244),$ $(453210, 000133, 003344),$ $(012345, 000333, 003344),$ $(012345, 000044, 002444),$ $(231045, 000124, 002244),$ $(453210, 000044, 001444)\}$

$\mathcal{S}_{hato,k}$

- $k = 1$: $\{(01, 00, 01),\ (10, 00, 01)\}$
- $k = 2$: $\{(012, 011, 022),\ (102, 000, 012),$ $(210, 002, 012)\}$
- $k = 3$: $\{(0123, 0000, 0133),$ $(1023, 0111, 0133),$ $(2310, 0002, 0133),$ $(3210, 0113, 0133)\}$
- $k = 4$: $\{(01234, 00222, 02244),$ $(12034, 00000, 01244),$ $(21034, 01111, 01244),$ $(34210, 00003, 01444),$ $(43210, 01114, 01444)\}$
- $k = 5$: $\{(012345, 000222, 013555),$ $(102345, 011333, 013555),$ $(231045, 000000, 013355),$ $(321045, 011111, 013355),$ $(453210, 000004, 013555),$ $(543210, 011115, 013555)\}$
- $k = 6$: $\{(0123456, 0022226, 0226666),$ $(1203456, 0111115, 0126666),$ $(2103456, 0000004, 0126666),$ $(3456210, 0000000, 0133666),$ $(4356210, 0111111, 0133666),$ $(5643210, 0002222, 0133666),$ $(6543210, 0113333, 0133666)\}$
